# Biochemical phenotype of a common disease-causing mutation and a possible therapeutic approach for the phosphomannomutase 2-associated disorder of glycosylation

**DOI:** 10.1002/mgg3.3

**Published:** 2013-03-27

**Authors:** Giuseppina Andreotti, Emilia Pedone, Assunta Giordano, Maria Vittoria Cubellis

**Affiliations:** 1 Istituto di Chimica Biomolecolare – CNRPozzuoli, Italy; 2 Istituto di Biostrutture e Bioimmagini – CNRNapoli, Italy; 3 Dipartimento di Biologia, Universita' Federico IINapoli, Italy

**Keywords:** Congenital disorders of glycosylation, glucose 1,6-bisphosphate, *N*-glycosylation, phosphomannomutase

## Abstract

Phosphomannomutase 2 (PMM2) deficiency represents the most frequent type of congenital disorders of glycosylation. For this disease there is no cure at present. The complete loss of phosphomannomutase activity is probably not compatible with life and people affected carry at least one allele with residual activity. We characterized wild-type PMM2 and its most common hypomorphic mutant, p.F119L, which is associated with a severe phenotype of the disease. We demonstrated that active species is the dimeric enzyme and that the mutation weakens the quaternary structure and, at the same time, affects the activity and the stability of the enzyme. We demonstrated that ligand binding stabilizes both proteins, wild-type and F119L-PMM2, and promotes subunit association in vitro. The strongest effects are observed with glucose-1,6-bisphosphate (Glc-1,6-P_2_) or with monophosphate glucose in the presence of vanadate. This finding offers a new approach for the treatment of PMM2 deficiency. We propose to enhance Glc-1,6-P_2_ concentration either acting on the metabolic pathways that control its synthesis and degradation or exploiting prodrugs that are able to cross membranes.

## Background

Mannose is required to build the glycan moieties that are added to proteins and other macromolecules. It is imported into the cytosol via transporter to be either isomerized into fructose or phosphorylated on C6. Mannose-6-phosphate (Man-6-P) is alternatively isomerized into fructose-6-phosphate by an isomerase (MPI) or converted into mannose-1-phosphate (Man-1-P) by a mutase. There are two paralogous mutases, which catalyze the reversible conversion of Man-6-P into Man-1-P in humans, phosphomannomutases 1 and 2 (PMM1 and PMM2). To a smaller extent, the same mutases catalyze the conversion of glucose 6-phosphate (Glc-6-P) into glucose 1-phosphate (Glc-1-P). They both require activation by glucose 1,6-bisphosphate (Glc-1,6-P_2_) or mannose 1,6-bisphosphate (Man-1,6-P_2_). They have also both been crystallized and their structures are deposited in the proteindatabank (PDB) (2fuc, 2fue, 2amy, 2q4r) (Silvaggi et al. 2006) (Levin et al. 2007). Man-1-P is needed to produce activated species, GDP-mannose and dolichol-phosphate-mannose, which are essential donor substrates for the assembly of the dolichol-linked oligosaccharide precursor by cytosolic and luminal oriented ER-mannosyltransferase. Thus, Man-1-P is a starting point for the synthesis of *N*-glycans, *O*-mannose linked glycans, glycophosphotidylinositol anchors, and C-mannosylated proteins; an impairment of its synthesis is the major cause of defects in the synthesis of glycoproteins or other glycoconjugates. The most common congenital glycosylation disorder (CGD) is in fact PMM2 deficiency (PMM2-CDG, MIM#212065), which was formerly known as Congenital Disorder of Glycosylation-Ia, CDG-Ia, or Jaeken syndrome; no CDG described so far is linked to mutations of PMM1. PMM2-CDG follows autosomal recessive inheritance and is a pan-ethnic rare disease although the carrier frequency for its most common mutation, p.R141H (rs28936415; NM_000303.2:c.422G>A; NP_000294.1:p.Arg141His), is rather high in certain populations (1/60–1/80 in Northern European population) (Schollen et al. 2000). Homozygosity for p.R141H has never been found because the protein is almost completely inactive in vitro and complete loss of the enzymatic activity of PMM2 is lethal (Kjaergaard et al. 1998; Matthijs et al. 1998). People carrying a single mutant allele are asymptomatic; people affected by PMM2-CDG have two mutant alleles and one of the mutations, at least, retains some enzymatic activity (Grunewald 2009). The occurrence of the disease is, therefore, determined by the incidence of hypomorphic mutations, which are rarer than p.R141H with a frequency of 1/300 or 1/400 in Northern European population (Schollen et al. 2000). The incidence of PMM2-CDG in the population is less than that expected on the basis of allele frequency. This discrepancy can be explained from reduced fertility in carriers, intrauterine death of affected fetuses, or underdiagnosis in postnatal life.

Although many therapeutic strategies are being evaluated for PMM2-CDG, there is still no cure for the disease. It was recently reported that prenatal mannose treatment in a hypomorphic mouse model could overcome damages to the embryos (Schneider et al. 2011). If this treatment did prove to be effective in humans, it would sensibly reduce intrauterine death, but unfortunately neither intravenous nor oral supplementation of mannose is effective for curing PMM2-CDG in postnatal life (Mayatepek et al. 1997; Mayatepek and Kohlmuller 1998). The use of morpholino oligonucleotides was proposed (Vega et al. 2009), but this cure would be very specific and only effective on splicing mutations. Other more general approaches have already been tested in vitro. One possibility is to inhibit the Man-6-P isomerase (Sharma et al. 2011), which competes for Man-6-P, and raise the intracellular concentration of the PMM2 substrate. Another one is to simply bypass PMM2 supplying the cells with hydrophobic derivatives of Man-1-P that are able to cross the cell membranes (Eklund et al. 2005). A new possibility of therapeutic intervention is offered by pharmacological chaperones that would increase the levels of PMM2 residual activity by stabilizing the mutant enzyme. Even a minor increment could be beneficial, as parents with 50% enzymatic activity have normal phenotype. Such a treatment has already been exploited for other genetic diseases like lysosomal storage disorders (Flanagan et al. 2009; Benito et al. 2011; Ishii 2012) or other metabolic disorders (Santos-Sierra et al. 2012). Its employment requires that the affected protein retains some activity and is only moderately unstable (Andreotti et al. 2010, 2011). As all PMM2-CDG patients carry at least one hypomorphic mutant form which must be able to fold and maintain residual enzymatic activity, all genotypes encountered in clinical practice could be responsive to pharmacological chaperones. A precise biochemical characterization of the mutant protein causing PMM2-CDG is needed as a single type of therapy might not be appropriate for all genotypes.

We started analyzing p.F119L (rs28936415; NM_000303.2:c.422G>A; NP_000294.1:p.Arg141His) because it is the second most frequent mutation encountered in PMM2-CDG patients (Matthijs et al. 1997). It is a very common mutation in Scandinavia, but it is widespread in many other countries of Northern Europe. Compound heterozygosity for p.R141H and p.F119L accounts for approximately one forth of a large cohort of patients examined from several countries (Matthijs 2000; Erlandson et al. 2001). Homozygosity for p.F119L was also observed in some PMM2-CDG patients. We extensively characterized both F119L-PMM2 and wild-type PMM2 (wt-PMM2) assessing their biochemical and enzymatic properties. We proved that the mutation produces several deleterious effects on the protein, reducing its activity and stability and affecting its quaternary structure. This explains why the clinical result on affected patients is on the severe end of the spectrum of PMM2-CDG (Kjaergaard et al. 2001). However, a precise knowledge of the phenotype of p.F119L at biochemical level allows to propose a new therapeutic approach for patients carrying this mutation or other severe ones of the same type. We identified molecules that stabilize the affected enzyme and improve its thermophilicity, thermostability, and resistance to proteases. These molecules can be used as lead compounds to develop pharmacological chaperones and to enhance enzyme activity.

## Methods

### Materials

Thermolysin from *Bacillus thermoproteolyticus rokko*, glucose-6-phosphate dehydrogenase from baker's yeast (*Saccharomyces cerevisiae*), phosphoglucose isomerase from rabbit muscle, phosphomannose isomerase from *Escherichia coli*, alpha-d-glucose 1-phosphate, d-glucose 6-phosphate, alpha-d-Glc-1,6-P_2_, alpha-d(+)Mannose 1-phosphate, beta-nicotinamide adenine dinucleotide phosphate were from Sigma-Aldrich (Milan, Italy). Sodium orthovanadate 99% was from Acros Organics (Geel, Belgium). Diethylaminoethyl (DEAE) Sepharose Fast Flow and Superdex-75 were from GE Healthcare Life Sciences (Milan, Italy).

### Cloning and mutagenesis

The complete open reading frame (ORF) encoding human wt-PMM2 was amplified using as a template the clone IMAGp958C172371Q, which contains the full length cDNA. Two primers PMM2_nh2(TGGGCATATGGCAGCGCCTGGCCCAG) and PMM2_cooh(CCGTTGGATCCTTAGGAGAACAGCAGTTC) were exploited to provide restriction sites to clone the ORF into a prokaryotic expression vector generating wt-PMM2-Pet22b+. A mutation, F119L, was introduced with two consecutive polymerase chain reactions (PCRs) using wt-PMM2-PET as a template. In the first round of amplifications, two reactions were set up, one contained the outmost forward oligo PMM2_nh2 and the specific reverse mutant oligo (CGGAATTCAATTAAAGTACCCCTCTTCTTCGGG) and the other contained the outmost reverse oligo (PMM2_cooh) and the forward specific mutant oligo (AGGGGTACTTTAATTGAATTCCGAAATGGGATG). In the second round of amplifications, the purified products of the first PCR reactions were used as templates and the outmost forward and reverse oligos were used as primers. The amplifications were performed for 28 cycles using the following conditions: 95°C for 10 min, 94°C for 30 sec, 60°C for 30 sec, 72°C 30 sec, and 72°C for 5 min with 0.6 μmol/L of each primer. After the second round, amplified fragments were purified, digested with NdeI and BamHI and inserted into Pet22b+ generating F119L-PMM2-Pet22b+. Recombinant plasmids were verified by sequencing.

### Protein expression and characterization

Both wt-PMM2 and F119L-PMM2 were expressed in *E. coli* BL21(DE3) strain grown at 37°C in Luria-Bertani broth containing ampicillin 0.2 mg/mL. The expression and purification of wt-PMM2 was performed as described (Pirard et al. 1999), with only minor changes.

The expression of F119L-PMM2 was assessed. The best production of the protein was obtained by adding IPTG 0.4 mmol/L when the optical density was 0.8 and prolonging the incubation for 4 h after induction. The cells were then harvested, washed with phosphate buffer saline (PBS), enzymatically lysed, and ammonium sulfate was added to the clear homogenate up to 60% saturation. The precipitate was recovered, redissolved in buffer, dialyzed against 4-(2-Hydroxyethyl)piperazine-1-ethanesulfonic acid, N-(2-Hydroxyethyl)piperazine-N′-(2-ethanesulfonic acid) (HEPES) 50 mmol/L pH 7.1, and loaded onto a DEAE-Sepharose ff column equilibrated with the same buffer. The pass-through was recovered, concentrated by ultrafiltration, and fractionated on a Superdex-75 column equilibrated with HEPES 20 mmol/L, NaCl 150 mmol/L, pH 7.1.

### Enzyme assays

When not differently specified, phosphomannomutase activity was assayed spectrophotometrically at 340 nm and 32°C by following the reduction of NADP+ to NADPH in 0.5 mL reaction mixture containing HEPES 20 mmol/L, pH 7.5, MgCl_2_ 5 mmol/L, NaCl 150 mmol/L, NADP+ 0.25 mmol/L in the presence of 0.6 mmol/L Man-1-P, 0.01 mg/mL yeast glucose 6-phosphate dehydrogenase, 0.010 mg/mL phosphoglucose isomerase, 0.0035 mg/mL phosphomannose isomerase, 0.03 mmol/L Glc-1,6-P_2_ (instead of Man-1,6-P_2_). Alternatively, phosphoglucomutase activity was measured in the same buffer in the presence of 0.6 mmol/L Glc-1-P, 0.03 mmol/L Glc-1,6-P_2_, and 0.01 mg/mL yeast glucose 6-phosphate dehydrogenase.

### Long-term stability

Long-term stability of F119L-PMM2 was investigated at 37 or 44°C under different conditions. The purified protein (0.027 mmol/L assuming molecular weight 28 kDa) was equilibrated in HEPES 50 mmol/L pH 7.1 containing NaCl 150 mmol/L with the specific ligands tested, EDTA 0.1 mmol/L, MgCl_2_ 5 mmol/L, MgCl_2_ 5 mmol/L plus Glc-1-P 0.5 mmol/L and vanadate 0.5 mmol/L or MgCl_2_ 5 mmol/L plus Glc-1,6-P_2_ 0.5 mmol/L.

At intervals up to approximately 6 h incubation time, aliquots containing 1.6 μg of F119L-PMM2 were taken and diluted immediately to assay the residual activity with Glc-1-P under standard conditions.

### Size exclusion chromatography

wt PMM2 (0.010 mg) and F119L-PMM2 (0.0065 mg) were subjected to size exclusion chromatography on BioSep-SEC-S3000 column (Phenomenex). The column had previously been equilibrated in HEPES 20 mmol/L pH 7.5, NaCl 150 mmol/L, MgCl2 5 mmol/L or in the same buffer containing Glc-6-P 0.5 mmol/L and vanadate 0.1 mmol/L. The chromatography was run at 0.5 mL/min on a HPLC system by Shimadzu and the absorbance at 280 nm was recorded.

### Light scattering

Wild-type PMM2 and F119L-PMM2 (ranging from 0.1 up to 0.5 mg in 0.025 mL) were injected and separated on a BioSep-SEC-S 3000 column (Phenomenex, Bologna, Italy) equilibrated at room temperature in HEPES 20 mmol/L pH 7.5 containing NaCl 150 mmol/L at 0.5 mL/min. To determine the effect of specific ligands (Mg^2+^ ions and Glc-6-P+ vanadate) on the quaternary structure of the proteins, gel filtration buffers were prepared containing 150 mmol/L NaCl with the addition of 5 mmol/L MgCl_2_ or Glc-6-P 0.5 mmol/L + MgCl_2_ 5 mmol/L + vanadate 0.1 mmol/L or EDTA 0.5 mmol/L. Light scattering data were recorded on an in-line miniDAWN™ TREOS triple-angle light scattering detector, and a Shodex RI-101 refractive index detector, supplied by Wyatt Technology Corporation. Data were analyzed by Astra 5.3.4.14 software (Wyatt Technology, Santa Barbara, CA) and fitted to the Zimm model with an estimated dn/dc value of 0.183 mL/g.

### Thermal stability

Heat-induced melting profile of wt-PMM2 and F119L-PMM2 was recorded by thermal shift assay, an assay which takes advantage of an environmentally sensitive fluorescent dye, Sypro Orange, or by circular dichroism.

Thermal shift assay was performed by using an iCycler iQ Real Time PCR Detection System (Bio-Rad, Hercules, CA). The proteins (0.2 mg/mL) were equilibrated in the presence of HEPES 20 mmol/L, NaCl 150 mmol/L, pH 7.5, Sypro Orange 2.5× (Invitrogen Molecular Probes, http://lifetechnologies.com).

The sample solutions were distributed in 96-well PCR plates (0.025 mL in each well), the plates were sealed with optical quality sealing tape, and heated from 25 to 80° at 0.5°C/min. The excitation wavelength of 490 nm and the emission wavelength of 575 nm, which are optimal for fluorescein, were adapted to detect Sypro Orange.

When the melting profile was obtained by circular dichroism (Jasco J-715 Circular Dichroism Spectrometer), the proteins (0.2 mg/mL) were equilibrated in HEPES 20 mmol/L, NaCl 150 mmol/L, pH 7.5 in the presence of different ligands.

The signal at 222 nm was recorded while temperature was increased at 0.5°C/min from 20 to 50, 55, 60, or 65°C accordingly to the melting temperature obtained by preliminary experiments.

The unfolded fraction was calculated as *f*_u_(*T*) = *f*(*T*) − *f*_n_(*T*)/*f*_d_(*T*) − *f*_n_(*T*) where *f* is the fluorescence or CD ellipticity at 222 nm at temperature *T*, *f*_n_(*T*), and *f*_d_(*T*) are the values of fluorescence or CD ellipticity at 222 nm extrapolated at temperature *T* from the native and unfolded regions of the melting profile. A Boltzmann model was used to fit the normalized fluorescence or molar ellipticity.

### Limited proteolysis

Purified wt-PMM2 and F119L-PMM2 were incubated (0.5 mg/mL) with thermolysin in HEPES 20 mmol/L, NaCl 150 mmol/L, MgCl_2_ 0.1 mmol/L, pH 7.5, at different protease: enzyme ratio (0, 1:150, 1:300, 1:600 w/w) for 1 or 2 h at 37°C in the presence of CaCl_2_ 9 mmol/L. Moreover, purified F119L-PMM2 was incubated (0.2 mg/mL) for 2 h at 37°C with thermolysin under the same conditions, in the presence with or without Glc-1,6-P_2_ 0.5 mmol/L, or Glc-6-P 0.5 mmol/L plus vanadate 0.5 mmol/L.

The reaction was stopped by addition of EDTA (40 mmol/L final concentration) and the samples (5 or 2 μg of each sample) were analyzed by SDS-PAGE (sodium dodecyl sulfate polyacrylamide gel electrophoresis). The protein bands were visualized by Coomassie blue staining and the images were acquired and the intensity of the bands quantified with a ChemiDoc XRS systems.

### Miscellaneous

Protein concentrations were routinely estimated using the Bio-Rad Protein System, with the bovine serum albumin as the standard. SDS-PAGE was performed using standard procedures (Laemmli 1970).Graph plotting and curve fitting were carried out with Kaleidagraph (Synergy Software, PA), unpaired *t*-test was carried out with Graphpad (La Jolla, CA).

## Results

### The enzymatic activity of PMM2 is influenced by its quaternary structure and by ligand binding

**Figure 1:**
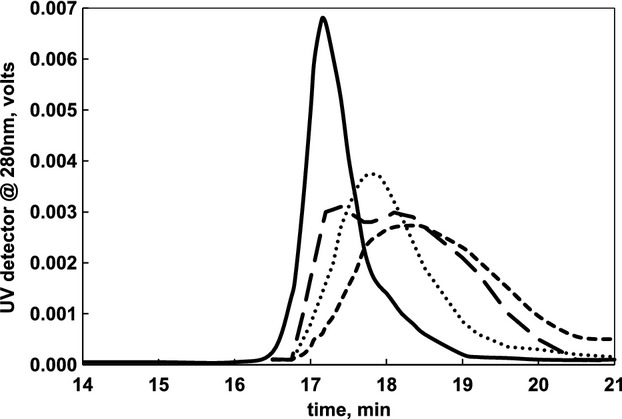
Ligand binding affects the quaternary structure of PMM2. Wild-type PMM2 (0.010 mg) and F119L-PMM2 (0.0065 mg) were subjected to size exclusion chromatography on BioSep-SEC-S3000 column equilibrated in HEPES 20 mmol/L pH 7.5, NaCl 150 mmol/L, MgCl_2_ 5 mmol/L (long dashed line for the wild-type PMM2 or short dashed line for F119L-PMM2) or in the same buffer containing Glc-6-P 0.5 mmol/L and vanadate 0.1 mmol/L (continuous line for wild-type PMM2 or dotted line for F119L-PMM2). The chromatography was run at room temperature at 0.5 mL/min.

The open reading frames of both wt-PMM2 and F119L-PMM2 were inserted into a prokaryotic expression vector without tag sequences. The recombinant proteins were obtained with a high yield (14 and 12 mg/L, respectively) and were purified with a protocol based on classic chromatographic methods similar to that used by Van Schaftingen and coworkers (Pirard et al. 1999). The homogeneity of the final products was proved with an SDS-PAGE which revealed a single band migrating at 28 kDa as expected for a polypeptide of 246 aa. Human PMM2 could only be assumed to be a dimer, as direct experimental evidence exists only for homologous species. Partially purified samples with phosphomannomutase activity extracted from rat liver, in fact, had been eluted in a gel filtration experiment with an apparent molecular weight of 60 kDa (Pirard et al. 1999).The crystal structure of the human enzyme deposited in the PDB contains only one subunit in the asymmetric unit; a possible biological assembly can be obtained using the PISA server (Krissinel and Henrick 2007). A weak interaction between subunits is most probable as only 7.6% of the subunit surface area becomes inaccessible to solvents when dimerized. Phenylalanine 119 is located at the interface between subunits and its mutation into Leucine, although conservative, would weaken the quaternary structure of the enzyme even more. This must be the case, because analytical size exclusion chromatography carried out in presence of Mg^2+^ reveals that wt-PMM2 has a smaller retention volume than F119L-PMM2 (Fig. 1). The molecular weights were calculated by multiangle light scattering; they are reported in Table 1. The value for wt-PMM2 is slightly lower than that expected for an homodimer formed by two subunits of 246 aa, the value for F119L-PMM2 is intermediate between the weight of a homodimer and that of a monomer. We suggest that there is an equilibrium for wt as well as for F119L-PMM2, which is shifted toward the monomer form for the mutant. The decrease of elution volumes of wt-PMM2 and of F119L-PMM2 recorded in the presence of Glc-6-P and vanadate, which is an inhibitor mimicking phosphate, demonstrates that ligand binding affects the quaternary structure and stabilizes the dimer further (Fig. 1). The observation that even for wt-PMM2 the monomeric form prevails in the presence of EDTA, confirms the dynamic quaternary association in PMM2 and the effect of ligands (Table 1).

**Table 1:** PMM2 molecular weights calculated by multiangle light scattering

	EDTA 0.5 mmol/L	MgCl_2_ 5 mmol/L	Glc-6-P 0.5 mmol/L + MgCl_2_ 5 mmol/L + vanadate 0.1 mmol/L
wt-PMM2	3.4 × 10^4^ (0.8%)	5.2 × 10^4^ (0.4%)	nd
F119L-PMM2	nd	3.4 × 10^4^ (2%)	5.1 × 10^4^ (1%)

Data were recorded on an in-line miniDAWN™ TREOS detector connected to a BioSep-SEC-S 3000 column. The chromatography was run at room temperature at 0.5 mL/min in HEPES 20 mmol/L pH 7.5 containing NaCl 150 mmol/L and the specified ligand. Data are shown as Da and percentage error is also indicated within parentheses.

We compared the phosphomannomutase activity of the mutant with that of the wt enzyme. Fitting saturation curves obtained varying Man-1-P, at 0.003 mg/mL total enzyme concentration and 3 μmol/L Man-1,6-P_2_, with Michaelis–Menten equation, under the simplistic assumption that Km and specific activity are constant, we found that *K*m is 11.8±1.0 μmol/L or 16.0±1.5 μmol/L and specific activity is 2.38±0.04 U/mg or 8.06±0.22 U/mg for F119L-PMM2 or wt-PMM2, respectively. Similarly, fitting curves obtained varying Glc-1-P, at 0.004 mg/mL total enzyme concentration and 30 μmol/L Glc-1,6-P_2_, we found that *K*m is 15.5±1.6 μmol/L or 8.2±1.4 μmol/L and specific activity is 1.40±0.04 U/mg or 3.34 ± 0.13 U/mg for F119L-PMM2 or wt-PMM2, respectively.

**Figure 2:**
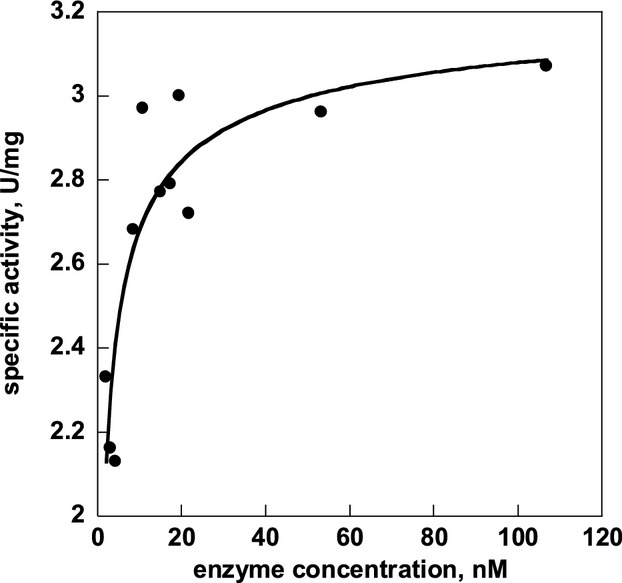
Specific activity of wt-PMM2 changes as a function of protein concentration. The assay was performed at 32°C in a reaction mixture containing HEPES 20 mmol/L, pH 7.5, MgCl_2_ 5 mmol/L, NaCl 150 mmol/L, NADP+ 0.25 mmol/L, Glc-1,6-P_2_ 0.030 mmol/L and yeast glucose 6-phosphate dehydrogenase 0.010 mg/mL. The reaction mixture also contained Glc-1-P 0.020 mmol/L and BSA 0.5 mg/mL. The wt-PMM2 concentration changed in the range 2–110 nmol/L (monomer equivalents).

**Figure 3:**
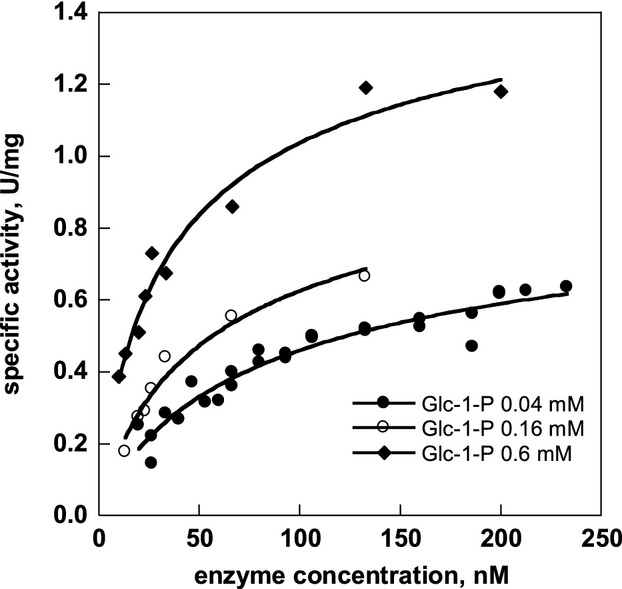
Specific activity of F119L-PMM2 depends on enzyme concentration. The assay was performed at 32°C in a reaction mixture containing HEPES 20 mmol/L, pH 7.5, MgCl_2_ 5 mmol/L, NaCl 150 mmol/L, NADP+ 0.25 mmol/L, Glc-1,6-P_2_ 0.030 mmol/L, and yeast glucose 6-phosphate dehydrogenase 10 μg/mL. The reaction mixture also contained BSA at 0.5 mg/mL. Three sets of experiments were carried out in the presence of 0.04, 0.16, or 0.6 mmol/L Glc-1-P and the F119L-PMM2 concentration changed in the range 10–240 nmol/L (monomer equivalents).


To use Man-1-P as a substrate, a coupled assay is needed which requires three different auxiliary enzymes (please see Methods for details). PMM2, however, is also active on Glc-1-P and can be tested for this activity with a simpler experimental procedure. A single auxiliary enzyme, Glc-6-P dehydrogenase (G6PD), consumes the product of phosphomannomutase, Glc-6-P, and produces NADPH which can be dosed spectrophotometrically. For this reason, we decided to monitor isomerization of Glc-1-P into Glc-6-P in the presence of 0.03 mmol/L Glc-1,6-P_2_ to assess the effect of enzyme concentration on the specific activity. The specific activity of wt-PMM2 calculated as a function of enzyme concentration (monomer equivalents), in the presence of 0.02 mmol/L Glc-1-P and 0.5 mg/mL BSA, is shown in Figure 2. The dependence of specific activity on enzyme concentration shown in Figure 2 suggests that the enzyme dimerizes and that the monomer has less activity than the dimer. With F119L-PMM2 three sets of experiments were carried out measuring specific activity as a function of enzyme concentration at different fixed levels of substrate, 0.6 mmol/L, 0.16 mmol/L or 0.04 mmol/L and 0.5 mg/mL BSA. In any case, we observed that its specific activity depends on the enzyme concentration too (Fig. 3). Like in the case of wt-PMM2, this can be explained assuming that there is an equilibrium between a dimer and a monomer: the latter is less active or inactive. To choose between these two possibilities, we used an approach suggested by Kurganov (1967). In the first place, we derived the specific activity of the dimer at each given concentration of Glc-1-P using the equation:

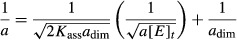
(1)

where *a* is the specific activity, [*E*]_t_ is the total concentration of enzyme (in monomer equivalents) *a*_dim_ is the specific activity of dimers, *K*_ass_ is the reciprocal of the dissociation constant *K*_dis_.

**Figure 4:**
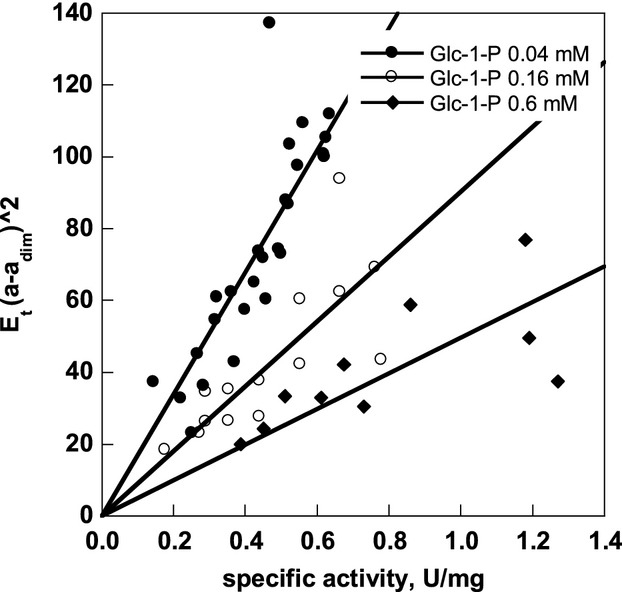
F119L-PMM2 monomer is inactive. Data from Figure 3 were fitted according to equation (1), where *a* is the specific activity, *a*_mon_ is the specific activity for the monomer, *a*_dim_ is the specific activity for the dimer, [E]t is the total concentration of enzyme (monomer equivalents).


Then, we replotted the data shown in Figure 3 and derived *a*_mon_, using the equation (2) and producing Figure 4.

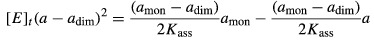
(2)

Straight lines passing through the origin resulted (Fig. 4). This showed that, within the limits of the experiment, the monomer is inactive.

Hence, to derive *K*_dis_ at any Glc-1-P concentration and to fit the data shown in Figures 2 and 3, we used equation (3) which assumes that the dimer is the only active species.


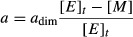
(3)

where [*M*] is the concentration of the inactive monomers, is given by


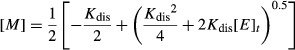
(4)

and is derived from the equilibrium condition


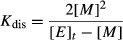
(5)

**Figure 5:**
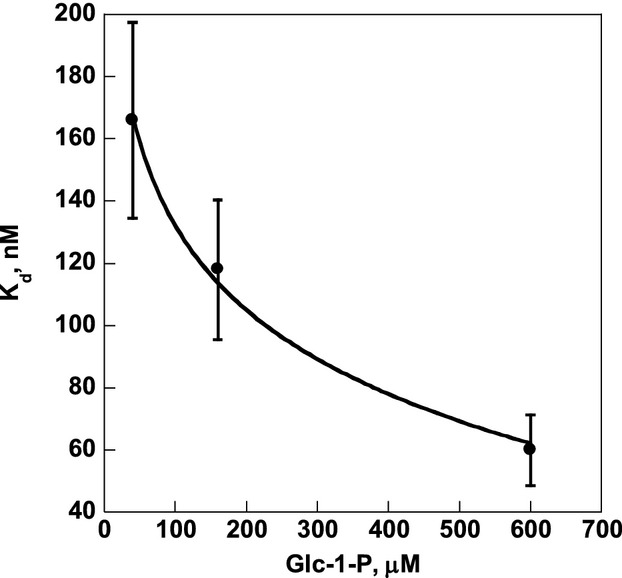
The dissociation constant for F119L-PMM2 dimer depends on substrate concentration. The dissociation constants, calculated from data of specific activity shown in Figure 3 and fitted to equation (2), were plotted as a function of the substrate concentration. The decay curve was obtained with logarithmic fitting and errors are shown by bars.

The dissociation constant for F119L-PMM2 dimer depends on substrate concentration as shown in Figure 5. Similarly, we could calculate a dissociation constant approximately 1–2 nmol/L varying the amount of wt-PMM2 (Fig. 2) at a fixed concentration of the substrate, Glc-1-P 0.02 mmol/L, which is comparable with estimated *K*m, 8.2±1.4 μmol/L. The dissociation constant of wt-PMM2 as well the maximal specific activity (3.27±0.20/3.30±0.20 U/mg) remain the same, within the experimental error, at high substrate concentration, Glc-1-P 0.6 mmol/L (data not shown).

*K*_dis_ of F119L-PMM2 is 100–150 fold higher (when it is measured at a substrate concentration, Glc-1-P 0.04 mmol/L, which is comparable to the estimated *K*m, 15.5±1.6 μmol/L) or 30- to 60-fold higher (when it is measured at high substrate concentration, Glc-1-P 0.6 mmol/L) than that of the wt. Hence, the stability of F119L-PMM2 dimers is lower than that of wt-PMM2, but the extent of the relative difference varies with substrate concentration.

In addition to this, using the equation (3) we extrapolated *a*_dim_, which is the specific activity of the dimers, at high substrate concentration, that is, Glc-1-P 0.600 mmol/L, and we calculated the true ratio between the activity of F119L-PMM2 and wt-PMM2 with Glc-1-P which is as high as 0.55 (1.8/3.3) under the condition employed (0.03 mmol/L Glc-1,6-P_2_).

**Figure 6:**
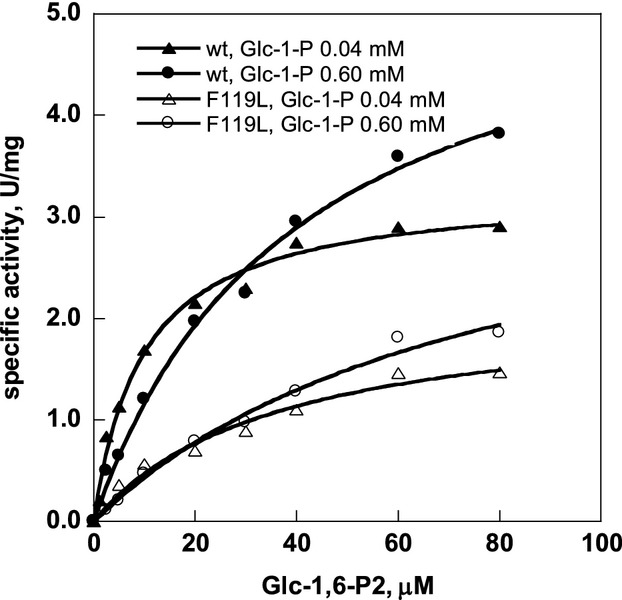
Specific activity of PMM2 depends on glucose-1,6-bisphosphate concentration. The assay was performed at 32°C in a reaction mixture containing HEPES 20 mmol/L, pH 7.5, MgCl_2_ 5 mmol/L, NaCl 150 mmol/L, NADP+ 0.25 mmol/L, Glu-1-P (0.04 or 0.60 mmol/L), and yeast glucose 6-phosphate dehydrogenase 10 μg/mL, while Glc-1,6-P_2_ was changed in the range 0–80 μmol/L. Enzymes concentrations were 107 nmol/L for wt-PMM2 and 73 nmol/L for F119L-PMM2. The hyperbolic dependence of velocity on the activator concentration was fitted using Michaelis and Menten equation to evaluate EC50.

Besides testing the effect of enzyme concentration, we tested the effect of Glc-1,6-P_2_ on wt-PMM2 and F119L-PMM2. We carried out experiments using variable concentrations of the activator at fixed enzyme concentration, 107 nmol/L for wt-PMM2 and 73 nmol/L for F119L-PMM2, and at fixed substrate concentration, 0.04 mmol/L or 0.6 mmol/L Glc-1-P (Fig. 6). The hyperbolic dependence of velocity on the activator concentration (Fig. 6) was fitted using Michaelis–Menten equation to evaluate the concentration at which Glc-1,6-P_2_ exerts half of its maximal effect. The EC50 depends on substrate concentration; at 0.04 mmol/L Glc-1-P, it is 10 or 36 μmol/L for the wt and the mutant enzyme, respectively, whereas at 0.6 mmol/L Glc-1-P, it is 39 or 78 μmol/L. In any case, the increasing Glc-1,6-P_2_ exerts a beneficial effect potentiating enzyme activity.

### Ligand binding stabilizes PMM2 against thermal induced denaturation

**Figure 7:**
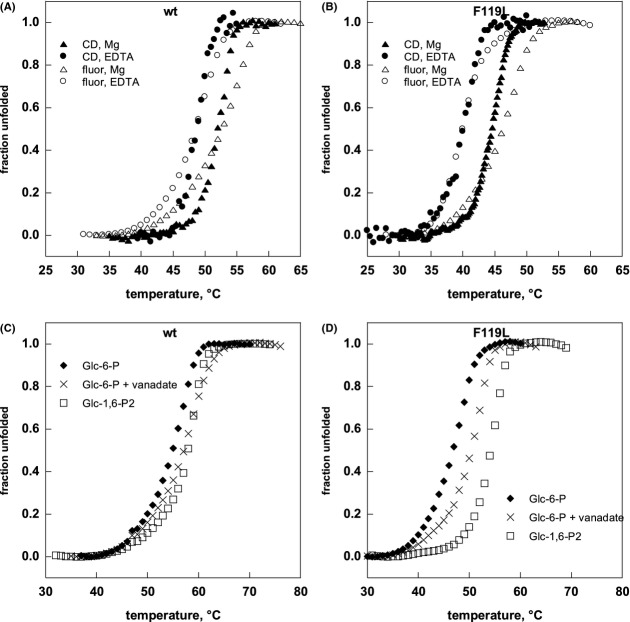
Ligand binding can affect the thermal stability of PMM2. Heat-induced melting profile of wild-type PMM2 (A and C) and F119L-PMM2 (B and D) were recorded by thermal shift assay and by circular dichroism. For thermal shift assay, the proteins (0.2 mg/mL) were equilibrated in buffer (HEPES 20 mmol/L, NaCl 150 mmol/L, pH 7.5) containing Sypro Orange2.5X and the appropriate ligands: MgCl_2_ 5 mmol/L, EDTA 5 mmol/L, Glc-6-P 0.5 mmol/L + MgCl_2_ 5 mmol/L, Glc-6-P 0.5 mmol/L + MgCl_2_ 5 mmol/L + vanadate 0.5 mmol/L, Glu-1,6-P 0.5 mmol/L + MgCl_2_ 5 mmol/L. The samples were distributed in 96-well PCR plates, the plates were sealed, and heated from 25 to 80° at 0.5°C/min. The experiment was run on an iCycler iQ Real Time PCR Detection System. An excitation wavelength of 490 nm and an emission wavelength of 575 nm were used to collect the data. When the melting profile was obtained by circular dichroism the proteins (0.2 mg/mL) were equilibrated in the same buffer in the presence of MgCl_2_ 1 mmol/L or EDTA 5 mmol/L. The signal at 222 nm was recorded while temperature was increased at 0.5°C/min from 20°C. The raw data were corrected by taking into account the slopes of the pre- and post-transition baselines, then they were normalized.

We assessed the influence of ligands on thermodynamic stability of PMM2 using thermal shift assay and CD. PMM2, both wild and mutant type, unfold irreversibly upon heating. For this reason, we evaluated the effect of different ligands carrying out all experiments at the same protein concentration (0.2 mg/mL) and scanning rate (0.5°C/min). We show the melting profile recorded by CD of wt-PMM2 in the presence of Mg^2+^ (dark triangles in Fig. 7A) or in the presence of EDTA (dark circles in Fig. 7A). The fraction unfolded increases from 10% to 90% over approximately 5–6°C in both cases, but the midpoint of the transition, *T*_0.5_, is shifted 3°C higher in the presence of the ion. The exposure of hydrophobic residues to the fluorescent dye recorded by thermal shift assay is shown with empty symbols in the same figure. It is less cooperative, but it confirms the strong stabilizing effect of Mg^2+^. As independent techniques produce different melting profiles, the unfolding of wt-PMM2 is not a two state process.

Under the same experimental conditions the midpoint temperature *T*_0.5_ of the mutant, measured either by CD (dark circles in Fig. 7B) or by thermal shift (empty circles in Fig. 7B) in the presence of EDTA are lower than those measured for wt-PMM2 (dark or empty circles in Fig. 7A), thus implying that the mutation destabilizes the enzyme. The stability of the mutant can be enhanced by ligand binding. The melting profile recorded by CD in the presence of the divalent ion (dark triangles Fig. 7B) is shifted toward high temperatures compared with the one recorded in the presence of EDTA (dark circles in Fig. 7B), with an increase of T_0.5_ of approximately 4°C. The fraction unfolded measured by CD increases from 10% to 90% over 5–6°C. In contrast with what observed with wt enzyme, the profiles recorded measuring molar ellipticity and fluorescence enhancement in the presence of EDTA (dark or empty circles Fig. 7B) are superimposable, suggesting the occurrence of a two state transition for the mutant. This simple model does not apply in the presence of Mg^2+^ (dark or empty triangles Fig. 7B) where the existence of an intermediate must be considered.

PMM2, mutant or wt, can be further stabilized. Glc-1-P and Glc-6-P produce a small, but statistically significant increase of the midpoint temperature with respect to that measured in the presence of Mg^2+^ only (diamonds in Fig. 5C and D and Table S1, *P*-values calculated with unpaired *t*-test are in supplementary files). Vanadate, does not enhance the stability of the apo-enzymes, but potentiates the effect of glucose monophosphates. The increase in *T*_0.5_ depends on the concentration of the ligands, but not on the position occupied by the phosphate (Table S1). We compared the effect of glucose and mannose derivatives on wt-PMM2 stability. Mannose monophosphates are slightly less effective than glucose mono-phosphates, but also their effect is potentiated by vanadate. A negative substituent on the ligand is needed, because mannose or glucose, even in the presence of vanadate, do not stabilize the enzyme (data not shown). As iminosugars are largely exploited as pharmacological chaperones for the symptomatic treatment of lysosomal storage diseases disorders (Flanagan et al. 2009; Benito et al. 2011; Ishii 2012), we also tested a natural analog of mannose, 1-deoxymannojirimycin hydrochloride, but also this derivative which does not carry a negative substituent, is ineffective, irrespective of the presence vanadate (data not shown). The strongest stabilization was observed with 1,6-bisphosphate sugars (Fig. 7C and D and Table S1). Glc-1,6-P_2_ is slightly more effective than Man-1,6-P_2_ (Table S1). It is worth observing that the effect can be even stronger with the mutant as observed in Figure 7C and D. Ligand stabilization measured by thermal shift assay is confirmed by experiments carried out by CD (Table S2).

### Ligand binding affects long-term stability and protease resistance of PMM2

**Figure 8:**
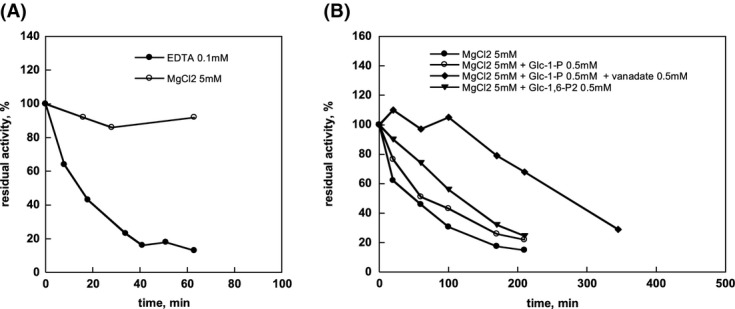
Long-term stability of F119L-PMM2. F119L-PMM2 (0.027 mmol/L of monomer equivalents) was equilibrated in HEPES 50 mmol/L pH 7.1 containing NaCl 150 mmol/L. Aliquots containing 1.6 μg of protein were taken at known incubation time and diluted immediately to assay the residual activity with Glc-1-P under standard conditions. (A) Results obtained at 37°C in the presence of EDTA 0.1 mmol/L or MgCl_2_ 5 mmol/L. (B) Results obtained at 44°C in the presence of MgCl_2_ 5 mmol/L, MgCl_2_ 5 mmol/L plus Glc-1-P 0.5 mmol/L, MgCl_2_ 5 mmol/L plus Glc-1-P 0.5 mmol/L and vanadate 0.5 mmol/L or MgCl_2_ 5 mmol/L plus Glu-1,6-P_2_ 0.5 mmol/L

Several factors including protein concentration, Mg^2+^, and temperature influence the stability of F119L-PMM2. Magnesium ions are necessary to keep F119L-PMM2 active at 37°C (Fig. 8A). Protein concentration affects F119L-PMM2 half-life because the diluted solutions of the mutant enzyme are rapidly inactivated even in the presence of Mg^2+^ and at 4°C; they are stabilized by the addition of BSA (data not shown). To test the effect of ligands on the half-life of the purified protein, we carried out experiments at relatively high protein concentration (0.027 mmol/L of monomer equivalents) at 44°C. At this temperature, the stability of the enzyme in the presence of Mg^2+^ decreases fast in the first hour of incubation and then relatively slowly. In the presence of ligands, the first decay is slowed down in particular in the presence of Glc-1,6-P_2_ and even more with Glc-6-P and vanadate (Fig. 8B).

**Figure 9:**
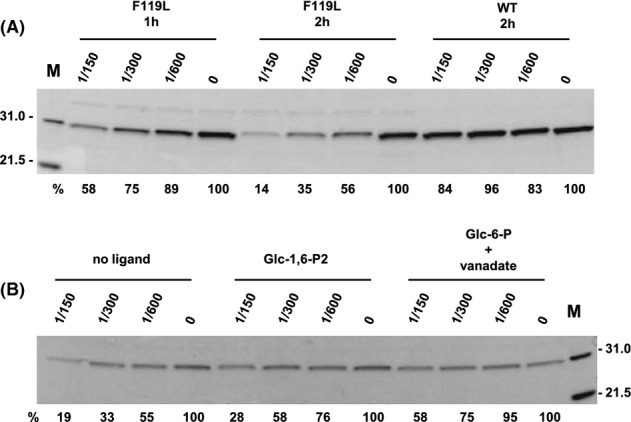
Ligand binding can increase the resistance to proteases of PMM2. Purified wild-type PMM2 and F119L-PMM2 (A) were incubated (0.5 mg/mL) with thermolysin in HEPES 20 mmol/L, NaCl 150 mmol/L, MgCl_2_ 0.1 mmol/L, pH 7.5 at the indicated protease substrate ratio (w/w) for 1 or 2 h at 37°C before they were analyzed (5 μg of each sample) by SDS-PAGE. Purified F119L-PMM2 (B) was incubated (0.2 mg/mL) with thermolysin in HEPES 20 mmol/L, NaCl 150 mmol/L, MgCl_2_ 0.1 mmol/L, pH 7.5 at the indicated protease substrate ratio (w/w), in the presence of no ligands, Glc-1,6-P_2_ 0.5 mmol/L or Glu-6-P 0.5 mmol/L plus vanadate 0.5 mmol/L, for 2 h at 37°C before they were analyzed (2 μg of each sample) by SDS-PAGE. The protein bands were visualized by Coomassie blue staining and the intensity of the bands quantified. The not-digested protein was quantified and expressed as percentage of the starting material (no protease panel A; time 0 panel B).

Enhancement in protein thermostability was often found to be associated with an increase in its resistance to proteases. To prove that this is the case for F119L-PMM2, we treated the enzyme with thermolysin at different protease/substrate ratios. Figure 9 shows that F119L-PMM2 is indeed less stable than the wt-PMM2 (panel A), but it becomes resistant to proteases in the presence of ligands (panel B).

## Discussion

The ultimate cause of most monogenetic disorders is the absence, scarcity, or the impairment of a given protein product. In order to cure the patients we should know which gene is altered but also which effect a specific mutation has on the protein. Mutations affecting the same gene can be grouped according to the effect they have on the protein, which we will define as the biochemical phenotype. Recent research has accumulated an enormous amount of genetic information, but very little about proteins. In some cases even the catalytic and thermodynamic properties of the wt enzyme have been poorly investigated like in the case of PMM2-CDG after the pioneering papers of Van Schaftingen and coworkers (Pirard et al. 1997, 1999). We proved that wt-PMM2 forms a weak dimer with a relatively small intersubunit buried surface. The dimer represents the active form of the enzyme. Magnesium ions as well as other ligands, substrates, activators, or inhibitors stabilize the enzyme. In particular, we found that mannose or glucose either 1,6-bisphosphate or monophosphate in the presence of vanadate stabilize wt-PMM2 thermodynamically and may represent useful lead compounds to design pharmacological chaperones for the symptomatic treatment of PMM2-CDG. In fact, ligands that are able to stabilize the wt form of an enzyme are usually able to stabilize mutant forms, provided that the mutants are folded and retain a functional binding pocket.

Not all the genotypes associated to a given monogenetic disease respond to pharmacological chaperones and a precise characterization of the mutants is needed to identify responsive ones. Mutations that affect the active site, such as p.R141H, are certainly excluded. We biochemically characterized p-F119L, the most frequent hypomorphic mutation observed in PMM2-CDG patients and one of the fewest which is observed in homozygosity. We confirmed that the mutant is active and we proved, using different techniques, that it is less stable than its wt counterpart. As predicted, the same ligands which stabilize wt-PMM2, are also able to stabilize the mutant enhancing its melting temperature, prolonging its resistance to high temperatures and to proteolysis. For this reason, in principle, patients affected by this mutation should benefit from the use of pharmacological chaperones. Freeze and coworkers (Sharma et al. 2011) proved that enhancing the concentration of the substrate Man-6-P is useful to improve *N*-glycosylation in the cells of patients affected by mild mutations, but it is not sufficient to raise the activity of enzyme in fibroblast of patients harboring the p.F119L mutation. We can confirm that Man-6-P as well as other mono-phosphate sugars have little stabilizing effect on wt-PMM2 as well as on F119L-PMM2. However, the effect of mono-phosphate sugars on thermostability and on quaternary structure is greatly potentiated by vanadate, an inhibitor which mimics phosphate and recreates a complex similar to sugar 1,6-bisphosphate in the active site. Although the substitution of F119 by L does not occur in the active site pocket, it causes a complex biochemical phenotype. In fact it affects the small intersubunit interface of the enzyme and pushes the equilibrium toward the monomeric form that has no activity. Moreover, it affects the maximal velocity and the specific activity of the dimer that is only half of that wt-PMM2. The complexity of the biochemical phenotype of F119L-PMM2 is reflected into the severity of clinical phenotype associated with this mutation and may require combined therapies. Even so a pharmacological chaperone would be beneficial to the patients as it would raise the intracellular concentration of the enzyme and favor subunit association. Usually pharmacological chaperones are reversible inhibitors of the affected enzyme, but there is no reason to exclude the usage of other types of molecules which might prove to be even more effective. These include allosteric ligands, cofactors, activators, or substrates. Glc-1,6-P_2_ has proved to be effective in enhancing enzymatic activity, thermal stability, and resistance to proteases. In addition to this, we observe that during catalysis, that is in the presence of the activator, an increase of the concentration of the substrate corresponds to a reduction of the dissociation constant of the dimer. Enhancement of Glc-1,6-P_2_ above the normal concentration would be beneficial in consideration of the fact that EC50 for F119L-PMM2 is relatively high.

Taken together these findings indicate that Glc-1,6-P_2_ represents a potential drug. How could we possibly increase its concentration in the cells? A few attempts have been carried out to produce hydrophobic derivatives of Man-1-P able to cross cell membrane (Rutschow et al. 2002). Freeze and coworkers (Eklund et al. 2005) neutralized the negative charges on the phosphate with acetoxymethyl groups and demonstrated that the protecting group, once hydrolyzed in the cell, generates nontoxic compounds. With a similar synthetic strategy it could be possible to produce a hydrophobic derivative of glucose or mannose-1,6-bisphosphate. As bisphosphates are activators of PMM2, they could be used at a lower concentrations with respect to Man-1-P.

Another possible strategy would be to enhance biosynthesis or to reduce degradation of the activators in the cells. Indeed Schaftingen and coworkers (Veiga-da-Cunha et al. 2008) demonstrated that it is possible to inhibit the enzyme which hydrolyzes Glc-1,6-P_2_ and increase the concentration of this metabolite in cells. Curiously this enzyme is PMM1 a paralogous enzyme which shares the mutase activity on Glc-6-P and Man-6-P with PMM2, but possess a specific phosphatase activity toward Glc-1,6-P_2_. PMM1 does not compensate for the loss of PMM2, its ablation in mice produces no obvious pathology or phenotype (Cromphout et al. 2006) and it has never been found associated to human pathologies. To treat PMM2-CDG patients it could be possible to exploit the fact that the specific phosphatase activity of PMM1 is enhanced 100 folds by inosine monophosphate (IMP) whereas the same nucleotide has little or no effect on PMM2 (Veiga-da-Cunha et al. 2008). Using this strategy it would be possible to inhibit PMM1, whose reduction has no deleterious effect apparently, without affecting PMM2. Indeed a few inhibitors have already been developed to inhibit IMP production by adenosine monophosphate (AMP) deaminase and some of them have an IC50 as low as 1–5 μmol/L (Skladanowski et al. 2005; Borkowski et al. 2008). This approach, is in principle useful for all hypomorphic PMM2 mutations, and, in particular, for those mutations which represent approximately 12% of all missense mutations found so far, that do not affect the binding pocket of PMM2, but occur between subunits. Frequent mutations, such as p.F119L and p.P113L (rs80338700, NM_000303.2:c.338C>T; NP_000294.1:p.Pro113Leu), belonging to this group are particularly harsh to treat and do not respond to other pharmacological approaches (Sharma et al. 2011). A molecule which activates and at same time stabilizes might represent the only possible therapy for mutants with a complex biochemical phenotype.

## Supporting Information

**Table S1.** Melting temperatures of wild-type PMM2 or F119L-PMM2 recorded by thermal shift assay.

**Table S2.** Melting temperatures of wild-type PMM2 and F119L-PMM2 recorded by circular dichroism.
