# Supplementary material for: Biochemical phenotype of a common disease-causing mutation and a possible therapeutic approach for the phosphomannomutase 2-associated disorder of glycosylation
Source: Mol Genet Genomic Med. 2013 Mar 27;1(1):32–44. doi: 10.1002/mgg3.3 (PMC3893156; doi:10.1002/mgg3.3)
Supplement: Supplementary file 1 [file mgg30001-0032-SD1.docx]

Table 1 Supplementary File

Melting temperatures of wild-type PMM2 or F119L-PMM2 recorded by thermal shift assay.

The enzymes were equilibrated in the presence of Hepes 20 mM, NaCl 150 mM, pH 7.5, Sypro Orange 2.5x and in the presence of the appropriate ligand. Midpoints of the thermal transitions, T_0.5 ,_ were defined by three parameter logistic fit and are reported in the third column, the differences of the same values with respect to the reference temperature measured in the presence of magnesium ions are reported in the fourth column. P-values are marked by asterisks, *<0.005, **<0.001,***<0.0005, ****<0.0001 and are shown in the fifth column.

| **PROTEIN** | **LIGAND** | **T_0.5_** | **ΔT_0.5_** | **p** |
| --- | --- | --- | --- | --- |
| wt-PMM2 | MgCl_2_ 5mM | 52.86±0.16 |  |  |
| wt-PMM2 | EDTA 5mM | 48.65±0.08 | -4.21±0.17 | **** |
| wt-PMM2 | Glc-6-P 0.2mM + MgCl_2_ 5mM | 53.60±0.08 | 0.73±0.17 | *** |
| wt-PMM2 | Glc-6-P 0.5mM + MgCl_2_ 5mM | 54.40±0.26 | 1.53±0.30 | **** |
| wt-PMM2 | Glc-6-P 0.2mM + MgCl_2_ 5mM + vanadate 0.2mM | 54.73±0.20 | 1.86±0.25 | **** |
| wt-PMM2 | Glc-6-P 0.5mM + MgCl_2_ 5mM + vanadate 0.5mM | 56.62±0.09 | 3.75±0.18 | **** |
| wt-PMM2 | Glc-1-P 0.2mM + MgCl_2_ 5mM | 53.41±0.21 | 0.55±0.25 | * |
| wt-PMM2 | Glc-1-P 0.5mM + MgCl_2_ 5mM | 54.17±0.19 | 1.31±0.24 | **** |
| wt-PMM2 | Glc-1-P 0.2mM + MgCl_2_ 5mM + vanadate 0.2mM | 54.10±0.19 | 1.24±0.24 | **** |
| wt-PMM2 | Glc-1-P 0.5mM + MgCl_2_ 5mM + vanadate 0.5mM | 55.62±0.17 | 2.76±0.23 | **** |
| wt-PMM2 | Glc-1.6-P 0.2mM + MgCl_2_ 5mM | 57.85±0.69 | 4.99±0.70 | **** |
| wt-PMM2 | Man-6-P 0.2mM + MgCl_2_ 5mM | 53.45±0.22 | 0.59±0.27 | * |
| wt-PMM2 | Man-6-P 0.5mM + MgCl_2_ 5mM | 54.00±0.18 | 1.14±0.23 | **** |
| wt-PMM2 | Man-6-P 0.5mM + MgCl_2_ 5mM + vanadate 0.5mM | 56.55±0.16 | 3.69±0.22 | ** |
| wt-PMM2 | Man-1-P 0.2mM + MgCl_2_ 5mM | 53.08±0.19 | 0.21±0.24 |  |
| wt-PMM2 | Man-1-P 0.5mM + MgCl_2_ 5mM | 53.69±0.04 | 0.83±0.15 | *** |
| wt-PMM2 | Man-1-P 0.5mM + MgCl_2_ 5mM + vanadate 0.5mM | 53.67±0.20 | 0.81±0.25 | *** |
| wt-PMM2 | Man-1.6-P 0.2mM + MgCl_2_ 5mM | 56.94±0.35 | 4.08±0.38 | **** |
| F119L-PMM2 | MgCl_2_ 5mM | 46.04±0.21 |  |  |
| F119L-PMM2 | EDTA 5mM | 39.83±0.32 | -6.21±0.38 | **** |
| F119L-PMM2 | Glc-6-P 0.2mM + MgCl_2_ 5mM | 45.95±0.08 | -0.1±0.20 |  |
| F119L-PMM2 | Glc-6-P 0.5mM + MgCl_2_ 5mM | 46.62±0.03 | 0.58±0.21 |  |
| F119L-PMM2 | Glc-6-P 0.2mM + MgCl_2_ 5mM + vanadate 0.2mM | 47.41±0.14 | 1.37±0.25 | **** |
| F119L-PMM2 | Glc-6-P 0.5mM + MgCl_2_ 5mM + vanadate 0.5mM | 50.13±0.10 | 4.08±0.23 | **** |
| F119L-PMM2 | Glc-1-P 0.2mM + MgCl_2_ 5mM | 46.18±0.12 | 0.14±0.2 |  |
| F119L-PMM2 | Glc-1-P 0.5mM + MgCl_2_ 5mM | 47.28±0.12 | 1.23±0.24 | **** |
| F119L-PMM2 | Glc-1-P 0.2mM + MgCl_2_ 5mM + vanadate 0.2mM | 47.10±0.14 | 1.06±0.25 | *** |
| F119L-PMM2 | Glc-1-P 0.5mM + MgCl_2_ 5mM + vanadate 0.5mM | 48.36±0.20 | 2.32±0.17 | **** |
| F119L-PMM2 | Glc-1.6-P 0.2mM + MgCl_2_ 5mM | 53.74±0.32 | 7.70±0.32 | **** |
| F119L-PMM2 | Glc-1.6-P 0.5mM + MgCl_2_ 5mM | 56.31±0.06 | 10.26±0.22 | **** |
