# Supplementary material for: Biochemical phenotype of a common disease-causing mutation and a possible therapeutic approach for the phosphomannomutase 2-associated disorder of glycosylation
Source: Mol Genet Genomic Med. 2013 Mar 27;1(1):32–44. doi: 10.1002/mgg3.3 (PMC3893156; doi:10.1002/mgg3.3)
Supplement: Supplementary file 2 [file mgg30001-0032-SD2.docx]

Table 2 (Supplementary files)

Melting temperatures of wild type PMM2 and F119L- PMM2 recorded by circular dichroism.

The proteins (0.2 mg/ml) were equilibrated in Hepes 20 mM, NaCl 150 mM, pH 7.5 in the presence of different ligands. Ellipticity at 222nm was recorded while temperature was increased at 0.5°C/min. The midpoint T_0.5_ of the thermal transition were defined with a three parameter logistic fit and are reported in the third column, the differences of the same values with respect to the reference temperature measured in the presence of magnesium ions are reported in the fourth column. P-values are marked by asterisks, *<0.005, **<0.001,***<0.0005, ****<0.0001 and are shown in the fifth column.

| **PROTEIN** | **LIGAND** | **T_0.5_** | **ΔT_0.5_** | **p** |
| --- | --- | --- | --- | --- |
| wt-PMM2 | MgCl_2_ 1mM | 51.93±0.06 |  |  |
| wt-PMM2 | EDTA 5mM | 48.76 ±0.08 | -3.18±0.10 | *** |
| F119L-PMM2 | MgCl_2_ 1mM | 44.47 ± 0.03 |  |  |
| F119L-PMM2 | EDTA 5mM | 40.04±0.06 | -4.43±0.07 | *** |
| F119L-PMM2 | Glc-1,6-P 0.5mM + MgCl_2_ 1mM | 55.13 ±0.32 | 10.66±0.32 | *** |
| F119L-PMM2 | Glc-6-P 0.5mM + MgCl_2_ 1mM + vanadate 0.1mM | 47.47 ±0.06 | 3.00±0.07 | *** |
